# Impact of equilibrative nucleoside transporters on *Toxoplasma gondii* infection and differentiation

**DOI:** 10.1128/mbio.02207-25

**Published:** 2025-09-30

**Authors:** Gabriel Messina, Amber Goerner, Charlotte Bennett, Euwen Brennan, Vern B. Carruthers, Bruno Martorelli Di Genova

**Affiliations:** 1 Department of Microbiology and Molecular Genetics, University of Vermont 169979, Burlington, Vermont, USA; 2 Department of Microbiology and Immunology, University of Michigan School of Medicine 242912, Ann Arbor, Michigan, USA; University of California Davis, Davis, California, USA

**Keywords:** *Toxoplasma gondii*, host-pathogen interactions, intracellular parasites, parasitology, nutrient transport

## Abstract

*Toxoplasma gondii* cannot synthesize purines *de novo* and must import them; however, the functional interplay among its four equilibrative nucleoside transporters (ENTs) homologs remains unclear. We systematically deconstructed this network by combining CRISPR-Cas9 knockouts with an auxin-inducible degron. Across all phenotypic assays, tachyzoite replication, nucleoside-analog sensitivity, alkaline-stress-induced differentiation, and murine cyst formation, the ΔTgENT2 strain was indistinguishable from the parental line, indicating that TgENT2 is dispensable under the conditions tested. In contrast, the double mutant ΔTgAT1ΔTgENT3 exhibited delayed bradyzoite differentiation *in vitro* and produced smaller brain cysts *in vivo*. This double deletion triggered approximately threefold transcriptional upregulation of TgENT1, whose product we partially localized to the plant-like vacuolar compartment (PLVAC). Conditional depletion of TgENT1 caused complete intracellular growth arrest, PLVAC swelling, and a purine-starvation-like transcriptomic program enriched for nucleoside phosphatases and cyclic-nucleotide phosphodiesterases. These findings reveal a compensatory salvage pathway in which the parasite reroutes purine acquisition through a vacuolar route when plasma-membrane import is compromised. Although this response sustains tachyzoite proliferation, it fails during the energetically demanding transition to bradyzoites, creating a metabolic bottleneck that impairs chronic infection. Our work reveals an adaptable yet ultimately limited purine-import network and identifies TgENT1, along with the vacuolar salvage axis it mediates.

**IMPORTANCE:**

In this manuscript, we demonstrate that *Toxoplasma gondii* employs a flexible transporter network that redirects to a vacuolar salvage route when primary transporters are compromised. Disrupting this backup pathway disrupts parasite growth, exposing an Achilles’ heel in purine homeostasis. Because nucleoside transporters are druggable, these findings suggest that the purine import machinery and TgENT1 may be potential targets for therapies against *T. gondii* infections.

## INTRODUCTION

*Toxoplasma gondii* is an obligate intracellular parasite that causes toxoplasmosis, a disease affecting approximately one-third of the global population and posing risks to immunocompromised individuals and developing fetuses. The parasite’s lifecycle includes acute (tachyzoite) and chronic (bradyzoite) stages, each characterized by distinct metabolic demands (1). A critical adaptation of *T. gondii* is its complete reliance on host-derived purines due to the absence of a *de novo* purine synthesis pathway (2). Understanding how *T. gondii* transports and manages purine resources is crucial for deciphering its survival strategies and identifying potential therapeutic targets. Equilibrative nucleoside transporters (ENTs) are key proteins involved in this purine acquisition from host cells (3). Here, we investigate the roles of four putative *T. gondii* ENTs: TgENT1 (TGME49_288540), TgENT2 (TGME49_500147), TgENT3 (TGME49_233130), and TgAT1 (TGME49_244440) across both acute and chronic stages, aiming to elucidate their distinct and overlapping functions in parasite survival and development.

*De novo* nucleic acid synthesis and salvage pathways are fundamental for cellular growth and survival, allowing cells to adapt to varying environmental conditions (4). Higher eukaryotes possess sophisticated mechanisms to modulate nucleoside transport, salvage, and metabolism, integrating intracellular and extracellular cues to maintain nucleotide homeostasis (5–9).

Many early branching eukaryotes have evolved complex mechanisms to adapt to fluctuating nucleoside availability (10–12). *T. gondii* exemplifies this adaptation through its complete dependence on host-derived purines, having lost the capacity for *de novo* purine synthesis during evolution (2, 11, 13). This reliance underscores the importance of understanding the mechanisms of purine acquisition in *T. gondii*. In host cells such as macrophages and neurons, purine levels are tightly regulated, presenting a challenging environment for the parasite (9, 14).

The parasite employs equilibrative nucleoside transporters (TgENTs) as its primary means of scavenging purines from the host (15). *T. gondii* utilizes two purine salvage pathways: the phosphorylation of adenosine by the enzyme adenosine kinase and the phosphoribosylation of purine nucleobases by hypoxanthine-guanine phosphoribosyltransferase (11). These pathways enable the parasite to salvage both adenosine and other purine nucleobases efficiently. Additionally, *T. gondii* possesses a functional *de novo* pyrimidine biosynthesis pathway, with uracil phosphoribosyltransferase facilitating pyrimidine salvage by converting uracil to uridine monophosphate (11).

The initial identification of ENT activity in *T. gondii* was demonstrated in extracellular tachyzoites, where adenosine, inosine, and hypoxanthine were transported into the parasite (16). Subsequent studies identified TgAT1 via insertional mutagenesis (17). Radioactive nucleoside incorporation assays revealed the presence of multiple nucleoside transporters in *T. gondii*, suggesting a complex system with at least one high-affinity adenosine transporter yet to be characterized (18). TgAT1 remains the only TgENT studied *in vitro* using heterologous expression systems (15).

In this study, we aim to elucidate the roles of TgAT1 and three other homologs, TgENT1, TgENT2, and TgENT3, across different developmental stages of *T. gondii*. We hypothesized that the network of purine transporters in *T. gondii* is characterized by functional redundancy and compensatory regulation, ensuring parasite survival under purine stress. To test this, we employed a systematic genetic approach to deconstruct this network by the investigation of the parasite’s adaptive response to each TgENT disruption and assess the importance of this transporter network for the critical developmental transition from the acute tachyzoite to the chronic bradyzoite stage. This strategy was designed to unmask the latent functions and regulatory connections within the purine acquisition machinery.

## RESULTS

### TgENT BLAST analysis

**Fig 1:**
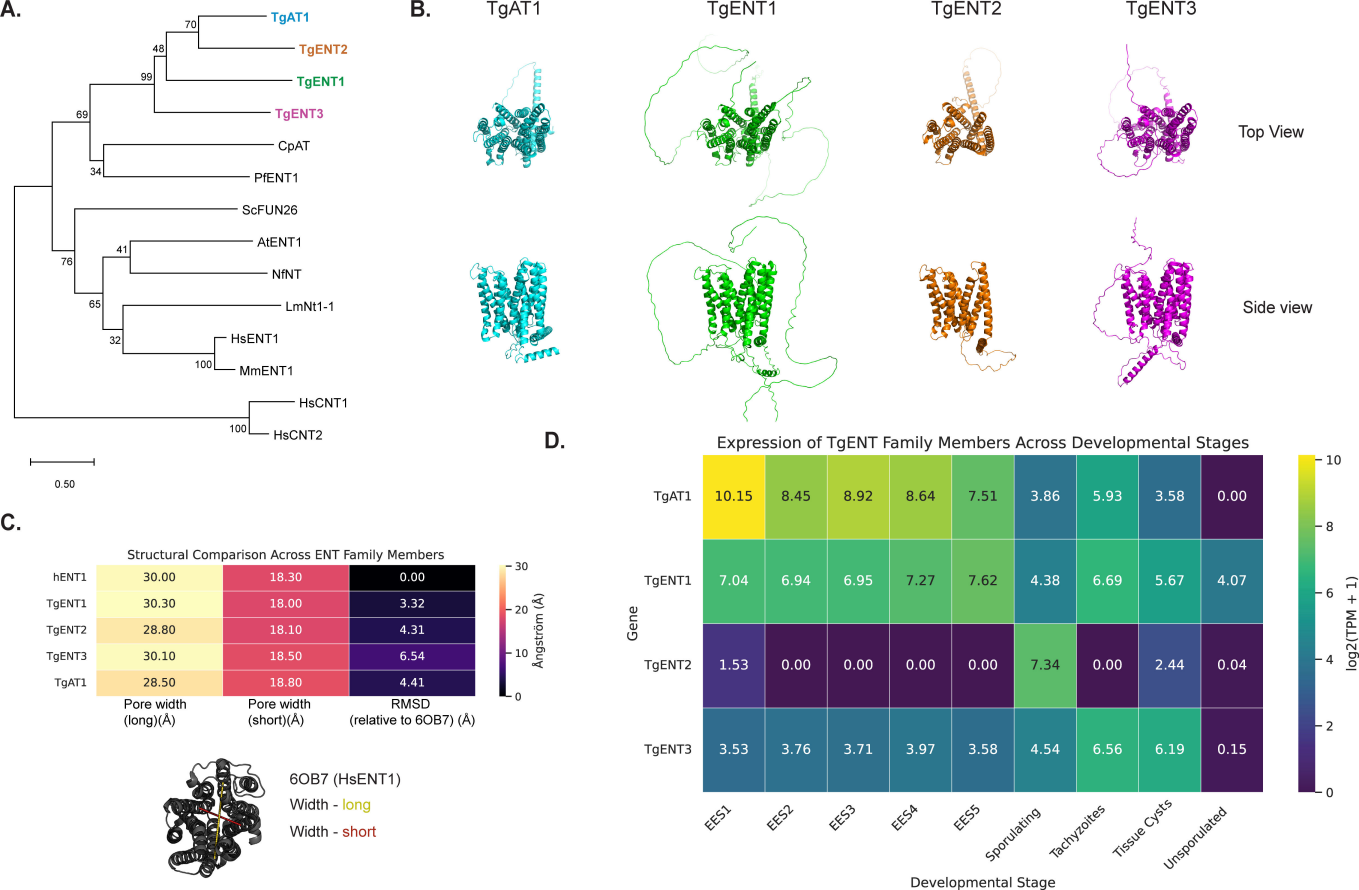
Evolutionary, structural, and transcriptional overview of the TgENT family. (**A**) Phylogenetic relationships: full-length protein sequences of the four *T. gondii* ENTs (TgAT1 and TgENT1–3) were aligned with representative ENT homologs from apicomplexan, fungal, plant, trypanosomatid, and mammalian lineages (CpAT, *Cryptosporidium parvum*; PfENT1, *Plasmodium falciparum*; ScFUN26, *Saccharomyces cerevisiae*; AtENT1, *Arabidopsis thaliana*; NNT, *Naegleria* sp.; LmNnt1-1, *Leishmania major*; HsENT1/2, MmENT1, human and mouse ENT1/2; HsCNT1/2, human concentrative nucleoside transporters—used as the out-group). MUSCLE-aligned sequences were subjected to neighbor-joining analysis; node labels give bootstrap support from 1,000 replicates (values <30% omitted). Scale bar, 0.5 substitutions per site. (**B**) Predicted tertiary structures: AlphaFold models of the four TgENT paralogs are shown in ribbon representation (top-view, upper row; side-view, lower row). Each adopts the canonical 11–13 trans-membrane helix bundle typical of ENT family members, with the central permeation pore clearly visible in the side view. Colors match the gene names in panel A. (**C**) Quantitative structural comparison to human ENT1 (PDB 6OB7). Pore dimensions were measured along the long and short axes (Å) for each TgENT model and for HsENT1, and Cα root mean square deviations (RMSDs) were calculated after SuperPose alignment to 6OB7. Heat-map shading reflects magnitude (scale bar, right). The cartoon underneath illustrates the pore-width measurements on the HsENT1 template. (**D**) Stage-specific expression of TgENT transcripts. Heat map of log2-transformed TPM values (+1) extracted from the ToxoDB developmental transcriptome. EES 1–5, enteroepithelial stages in cat epithelium; sporulating oocysts; tachyzoites; tissue cyst bradyzoites; unsporulated oocysts (19, 20). TgAT1 is highest in enteroepithelial and early tachyzoite stages, TgENT1 is broadly expressed, TgENT2 peaks during sporulation, and TgENT3 shows moderate expression throughout.

We performed protein-protein BLAST or position-specific iterated BLAST on NCBI using Human ENT-1 (3) and *Plasmodium falciparum* PfENT1 (12) nucleoside transporters as query sequences to search for *T. gondii* sequences with high similarity. Phylogenetic analysis (Fig. 1A) places TgAT1 and TgENT1–3 in a single, well-supported clade with apicomplexan and mammalian ENTs. Conserved-domain searches and AlphaFold modeling suggest that TgAT1 and TgENT1-3 harbor the hallmark 11-ENT fold with an open central pore (Fig. 1B and C; Table 1). Pairwise structural comparisons to the HsENT1 crystal structure (PDB 6OB7) show pore dimensions within the expected range for functional ENT homologs (Fig. 1C), supporting their candidacy as bona fide transporters. Finally, re-analysis of the transcriptomic data (19, 20) (Fig. 1D) reveals distinct but overlapping expression patterns; TgAT1 and TgENT1 are abundant throughout enteric, tachyzoite, and bradyzoite stages, TgENT3 is moderately expressed across the life cycle, and TgENT2 peaks during sporulation, suggesting stage-specific expression. A multiple-sequence alignment of TgENT1, TgENT2, TgENT3, TgAT1, and other characterized ENT homologs from different organisms is provided in Fig. S1.

**TABLE 1:** BLAST results for *T. gondii* sequences with high similarity to human ENT1 and *Plasmodium falciparum* ENT1

Gene ID	Name	Localization by hyperLOPIT (21)	PFam ID	PFam description	Phenotype score (22)
TGME49_233130	TgENT3	PM integral, Golgi	PF01733	ENT	0.04
TGME49_244440	TgAT1	PM integral, Golgi	PF01733	ENT	1.02
TGME49_288540	TgENT1	None	PF01733	ENT	−3.68
TGME49_500147	TgENT2	None	PF01733	ENT	0.47

### Nucleoside analogs growth assay

We hypothesized that the deletion of genes encoding nucleoside transporter homologs would negatively affect parasite growth. However, none of the single knockout strains displayed significant differences in either short-term or long-term growth rates compared to the parental strain (Fig. S2). The growth phenotype of TgENT1 knockdown parasites is described separately below (Fig. 4).

**Fig 2:**
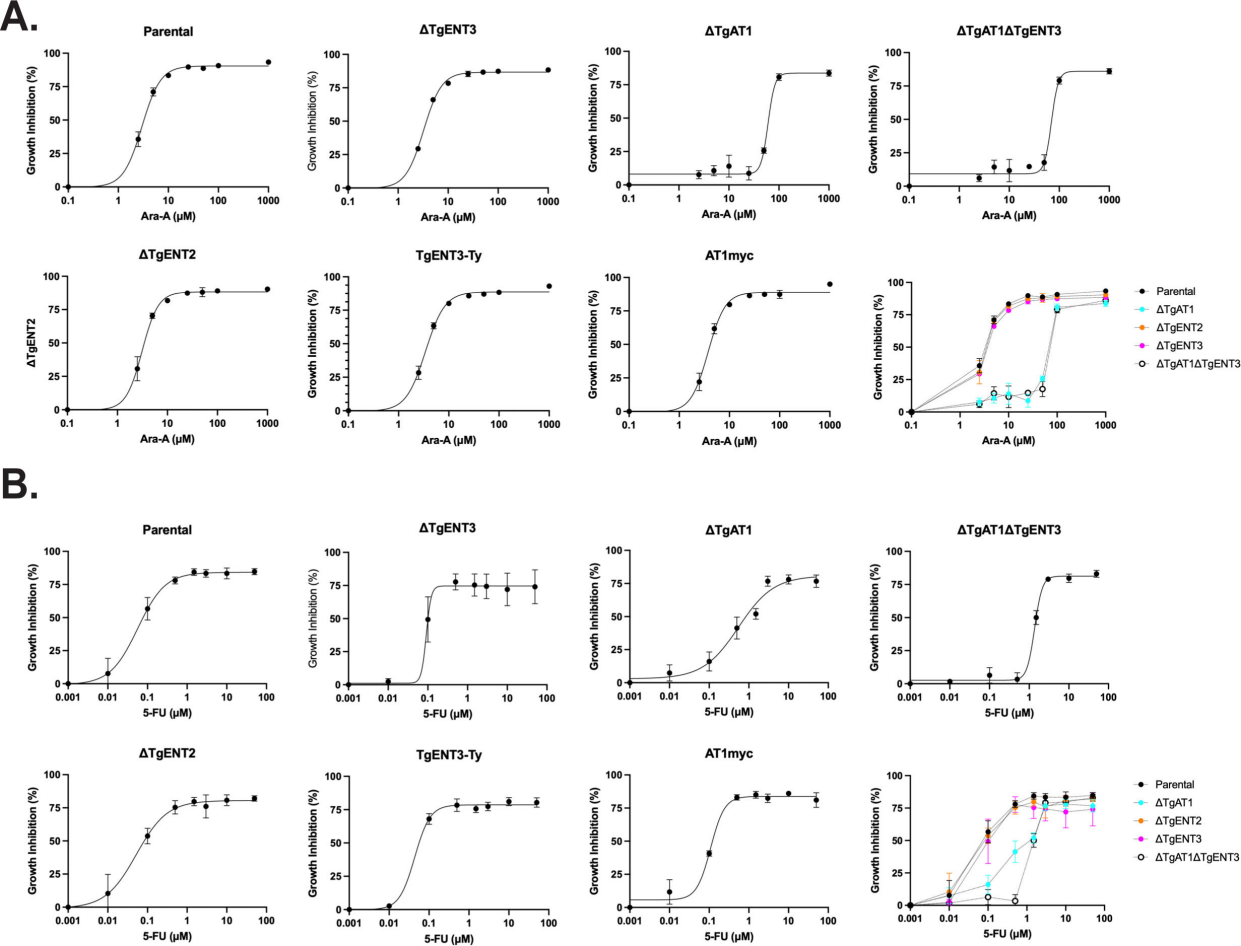
Deletion of TgAT1 and TgENT3 alters *Toxoplasma* sensitivity to toxic nucleoside analogs. Parental ME49 parasites and the indicated mutants were allowed to replicate for 72 h in Hs27 monolayers in the presence of increasing concentrations of (**A**) the adenosine analog Ara-A or (**B**) the pyrimidine analog 5-FU. Cultures were fixed and stained with anti-*Toxoplasma* (anti-Tg) antibody, and the mean area of ≥50 PVs per condition was measured by automated image analysis. Growth inhibition (% ±SEM) was calculated relative to drug-free controls and fitted to a four-parameter logistic curve (GraphPad Prism). Data are from three biological replicates, each performed in technical triplicate. Panel layout: for each drug, individual dose–response curves are shown for the parental line, single knockouts (ΔTgENT3, ΔTgENT2, and ΔTgAT1), the double knockout (ΔTgAT1ΔTgENT3), and the corresponding epitope-tagged complementation lines (ΔTgENT3::TgENT3-Ty1, ΔTgAT1::TgAT1-myc). The right-most graph in each row overlays all strains for direct comparison (color key, inset). IC_50_ values for each strain are shown in Table S1.

Among the four ENT homologs in *T. gondii*, only TgAT1 has been biochemically characterized (15). To determine whether deletion of TgENT2, TgENT3, or TgAT1 alters parasite sensitivity to the toxic nucleoside analogs 9-β-D-arabinofuranosyladenine (Ara-A) (18) and 5-fluorouracil (5-FU) (23), we grew the respective knockout strains in 96-well plates for 3 days in the presence of increasing concentrations of each analog. Infected monolayers were then fixed and stained with a rabbit anti-*Toxoplasma* (anti-Tg) antibody. Using immunofluorescence microscopy, we measured the area of at least 50 parasitophorous vacuoles (PVs) per condition; the mean PV size served as a quantitative read-out of growth inhibition (Fig. 2).

Consistent with TgAT1’s role as a purine transporter (15), ΔTgAT1 parasites showed increased resistance to the adenosine analog Ara-A (Fig. 2A). Unexpectedly, ΔTgAT1 also exhibited resistance to the pyrimidine analog 5-FU (Fig. 2B), suggesting broader substrate specificity than previously recognized. In contrast, the ΔTgENT2 strain did not differ from the parental line (Fig. 2), likely because TgENT2 is not expressed during the tachyzoite stage (Fig. 1D). Similarly, the ΔTgENT3 single knockout showed no difference in sensitivity relative to the parental line under drug treatment (Fig. 2). However, the double knockout ΔTgAT1ΔTgENT3 formed significantly larger PVs than ΔTgAT1 under 10 µM Ara-A, indicating that deletion of TgENT3 further enhances resistance to this analog. Conversely, ΔTgAT1ΔTgENT3 vacuoles were significantly smaller than those of ΔTgAT1 under 5 µM 5-FU, although they remained larger than parental vacuoles. Collectively, these observations suggest that while TgENT3 deletion may confer increased resistance to Ara-A in the ΔTgAT1ΔTgENT3 background, it also leads to heightened sensitivity to 5-FU.

### Role of TgENTs homologs in chronic infection

We successfully generated knockout strains for TgAT1, TgENT2, and TgENT3 using CRISPR/Cas9-mediated gene disruption in the ME49 background. Multiple attempts to generate a TgENT1 knockout strain were unsuccessful, suggesting that this gene may be essential for parasite viability. This prompted us to instead use the mini auxin-inducible degron (mAID) system for conditional knockdown analysis (Fig. 4) in the RH background.

Based on published transcriptomic data (19, 20) (Fig. 1D), TgENT1, TgENT3, and TgAT1 are constitutively expressed throughout the parasite life cycle, whereas TgENT2 expression is restricted to bradyzoite and sexual developmental stages, explaining the lack of phenotype in tachyzoite growth assays. Given this distinct expression pattern, as well as the unexpected lack of growth defects in single knockout strains, we next investigated potential changes in cyst formation using an *in vitro* and *in vivo* model.

**Fig 3:**
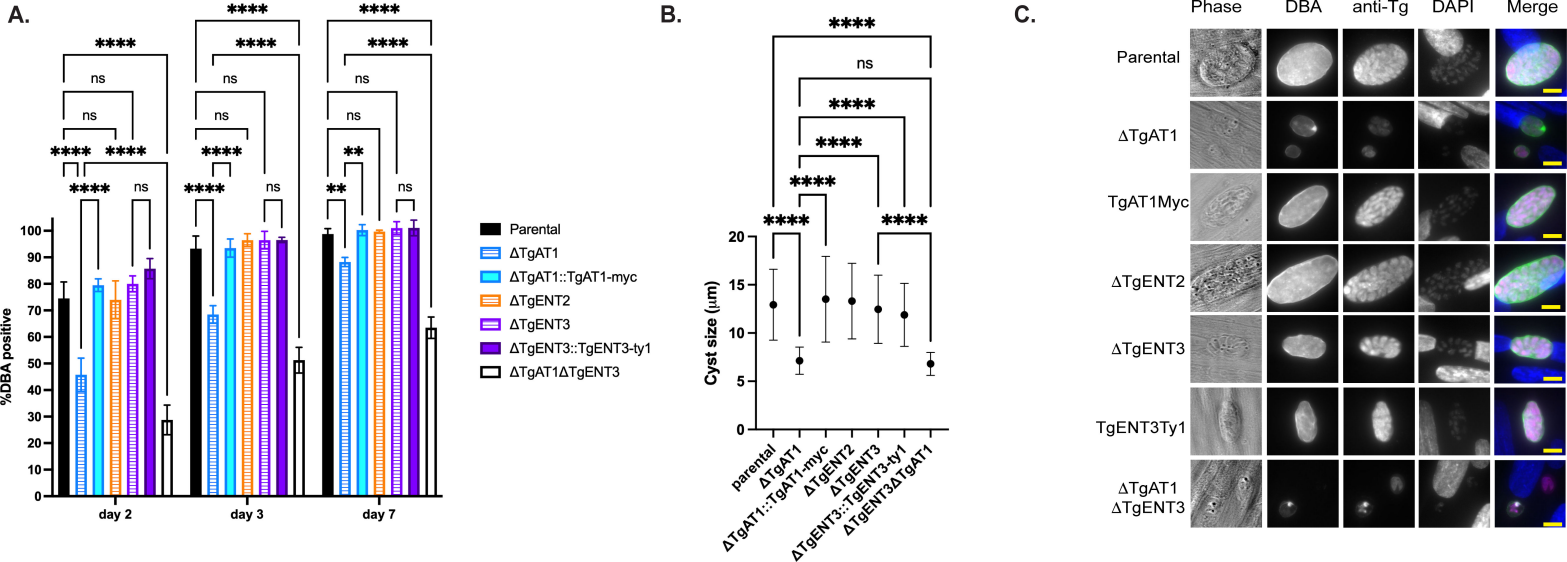
Simultaneous deletion of TgENT3 and TgAT1 delays differentiation. (**A**) Percentage of differentiation of the parental line, single knockouts (ΔTgENT3, ΔTgENT2, and ΔTgAT1), the double knockout (ΔTgAT1ΔTgENT3), and the corresponding epitope-tagged complementation lines TgENT3-Ty1 (ΔTgENT3::TgENT3-Ty1) and TgAT1-myc (ΔTgAT1::TgAT1-myc) at days 2, 3, and 7 post-invasion of Hs27 monolayers cultured in alkaline stress conditions (pH 8.1 and atmospheric CO_2_). Parasites were stained with immunofluorescent antibodies 4′,6-diamidino-2-phenylindole (DAPI), anti-Tg, and DBA. Bars show the percentage of DBA-positive vacuoles (thresholds set based on parental line intensity) over total vacuoles (anti-Tg). Slides were imaged with BioTEK Cytation 7 and quantified using Gen5 software (≥100 vacuoles were counted per replicate, *n* = 3 biological replicates). Statistical significance was assessed using one-way analysis of variance (ANOVA) (****P < 0.0001, ns = non-significant). (**B**) Average sizes of each cyst (DBA-positive vacuole) on day 7 postinvasion. (**C**) Representative images of vacuoles from each parasite line. Scale bar, 10 µm.

We assessed the impact of TgENT deletion on parasite differentiation by culturing infected human foreskin fibroblasts (Hs27) monolayers cultured in RPMI medium adjusted to pH 8.2 and atmospheric CO_2_. Monolayers were fixed after 2, 3, or 7 days, and differentiation rates were quantified using immunofluorescence assays (IFAs) with the lectin *Dolichos biflorus* Agglutinin (DBA), which stains *T. gondii’s* cyst wall (24). Parasites were co-stained with anti-Tg antibody, which identifies both tachyzoites and bradyzoites, to quantify all vacuoles in the monolayers and calculate differentiation ratios among strains. ΔTgAT1 and ΔTgAT1ΔTgENT3 parasites exhibited significantly slower differentiation. Both ΔTgAT1 and ΔTgAT1ΔTgENT3 have delayed differentiation, exemplified by a lower number of DBA vacuoles in the population (Fig. 3A). Moreover, after 3 days in pH 8.2 medium, the DBA-positive vacuoles formed by ΔTgAT1 and ΔTgAT1ΔTgENT3 parasites are smaller than those of parental and complement strains (Fig. S3). By day 7, only 88% of ΔTgAT1 and 63% of ΔTgAT1ΔTgENT3 parasites were DBA-positive, compared to approximately 99% in the parental and ΔTgENT3 strains (Fig. 3A). As we noticed at day 3, the ΔTgAT1 and ΔTgAT1ΔTgENT3 DBA-positive vacuoles are significantly smaller at day 7 as well compared to other strains (Fig. 3B). While ΔTgENT3 did not show a reduction in differentiation rate, the accentuated phenotype in the double deleted strain suggests an interplay between TgAT1 and TgENT3 in parasite differentiation under alkaline stress.

To assess long-term differentiation *in vivo*, male C57BL/6 mice were intraperitoneally infected with 250 parasites. Four weeks post-infection, mice were euthanized in accordance with Institutional Animal Care and Use Committee (IACUC) guidelines. Brains were extracted surgically, homogenized in phosphate-buffered saline (PBS) through sequential passage using 18-, 20-, and 22-gage needles, fixed, and stained with DBA. Cyst numbers and sizes were quantified for each strain, as previously done (25) (Fig. S4). This experiment was independently replicated twice, with four mice per strain in each replicate. No significant differences in cyst counts were observed among the strains (Fig. S4A). These findings suggest that, despite initially impaired differentiation observed in ΔTgAT1 and ΔTgAT1ΔTgENT3 parasites, these mutants ultimately compensate for this deficit during chronic infection in mice. The cyst sizes for ΔTgENT3 and ΔTgAT1ΔTgENT3 were significantly reduced compared to all other strains, suggesting that TgENT3 may have important roles during bradyzoite replication (Fig. S4B).

**Fig 4:**
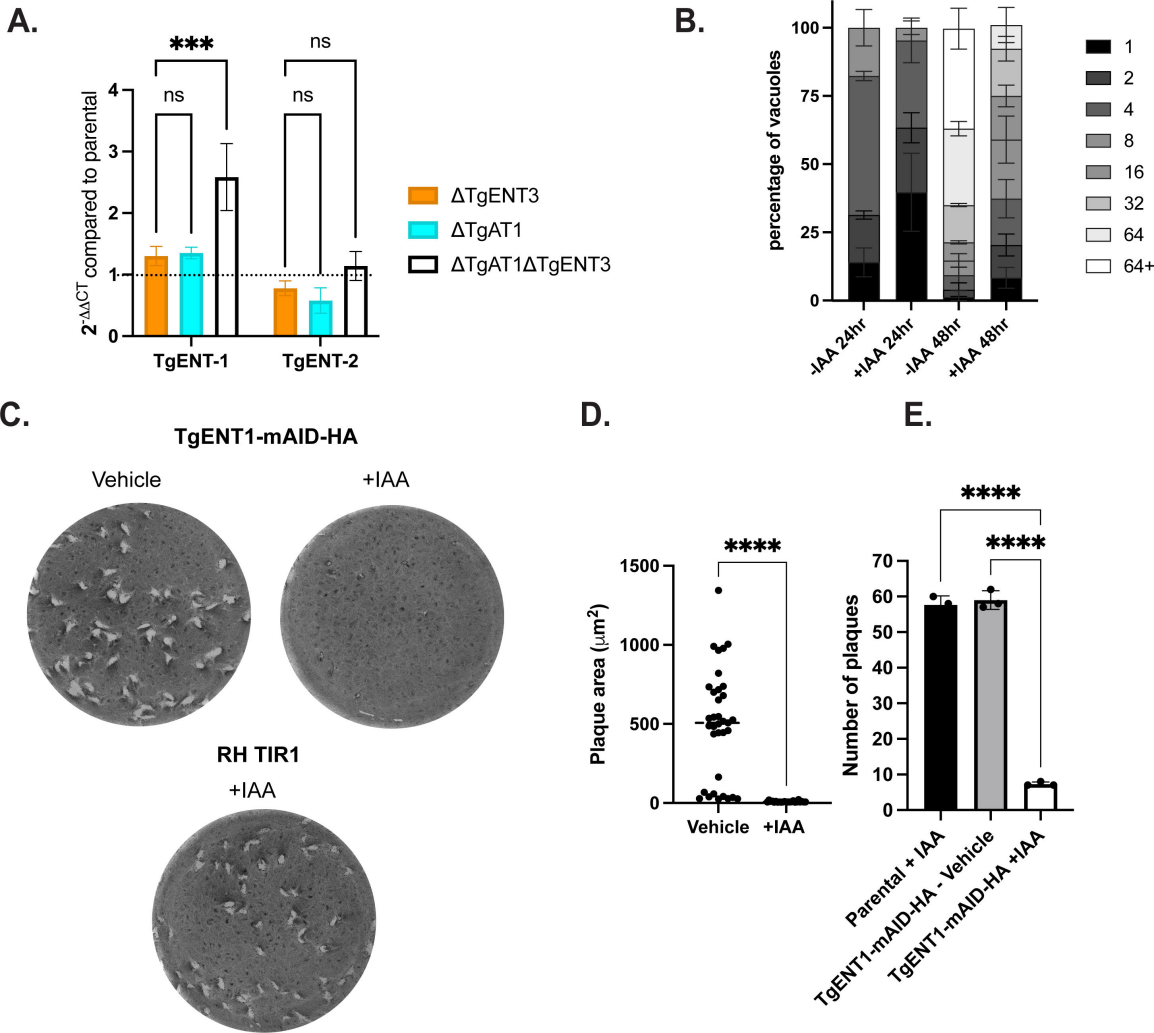
TgENT1 is transcriptionally upregulated in ΔTgAT1ΔTgENT3 parasites and is indispensable for intracellular replication and plaque formation. (**A**) qRT-PCR analysis of compensatory TgENT expression. Total RNA from tachyzoites of the indicated mutant lines was analyzed by qRT-PCR with primers specific for TgENT1 and TgENT2. Bars show 2^ΔΔCt^ values (mean ± SEM, *n* = 3 biological replicates) normalized to the parental line (dotted line = 1). Only the double knockout (ΔTgAT1ΔTgENT3, open bars) exhibits approximately threefold induction of TgENT1 (one-way ANOVA with ***P < 0.001); TgENT2 is unchanged (ns). (**B**) Acute intracellular growth after TgENT1 depletion. TgENT1-mAID-HA parasites were pre-treated for 48 h with vehicle or 500 µM indole-3-acetic acid (+IAA) to trigger auxin-inducible degradation, then allowed to invade fresh Hs27 monolayers for a further 48 h under the same conditions. Stacked bars depict the distribution of vacuoles containing 1, 2, 4, 8, 16, 32, 64, or >64 parasites (≥50 vacuoles counted per replicate; three replicates). (**C**) Plaque-formation assay. Wells were inoculated with 50 tachyzoites of the indicated strains and cultured for 9 days ± 500 µM IAA before fixation and crystal-violet staining. Representative wells are shown. (**D**) Quantification of plaque area. Individual plaque areas from TgENT1-mAID-HA wells (vehicle vs +IAA) are plotted (median ± inter-quartile range; *n* = 15–20 plaques). Student *t*-test, ****P < 0.0001. (**E**) Quantification of plaque number. Mean ± SEM plaque counts per well from three independent experiments. *t*-test, ****, *P* < 0.0001.

To investigate whether the comparable tachyzoite growth rate (Fig. S2) observed in ΔTgAT1ΔTgENT3 was due to compensatory expression of another TgENT family member, we performed quantitative PCR (qPCR) analyses. We compared the expression levels of the remaining TgENT transporters, TgENT1 and TgENT2, across tachyzoites derived from ΔTgAT1ΔTgENT3, single knockout strains, and the parental strain. qPCR analysis revealed approximately threefold upregulation of TgENT1 transcripts specifically in ΔTgAT1ΔTgENT3 parasites compared to parental controls (*P* < 0.05), while TgENT2 expression remained unchanged (Fig. 4A). Notably, single knockout strains, ΔTgAT1 and ΔTgENT3, showed no compensatory changes in TgENT1 or TgENT2 (Fig. 4A). These data indicate that the absence of both TgAT1 and TgENT3 may trigger compensatory upregulation of TgENT1, maintaining tachyzoite growth rates similar to single knockout strains.

### TgENT1 conditional deletion results in complete growth arrest

TgENT1 has a markedly negative genome-wide CRISPR phenotype score of –3.68 (22). On this scale, increasingly negative values typically denote progressively stronger fitness costs, underscoring TgENT1’s critical importance for parasite survival (Table 1). To investigate the role of TgENT1, we developed a parasite line, TgENT1-mAID-HA, in the RH background with regulated TgENT1 expression via 3-indoleacetic acid (IAA) treatment (26). We assessed the impact of TgENT1 depletion on parasite growth by culturing the parasites with IAA for 48 h. The treated parasites exhibited reduced replication when invading new Hs27 monolayers (Fig. 4B and C), indicating that TgENT1 expression is crucial for normal growth and replication. Additionally, the observation of reduced plaque size/plaquing efficiency evidences a long-term growth defect (Fig. 4D).

### TgENT1 localizes to the PLVAC

**Fig 5:**
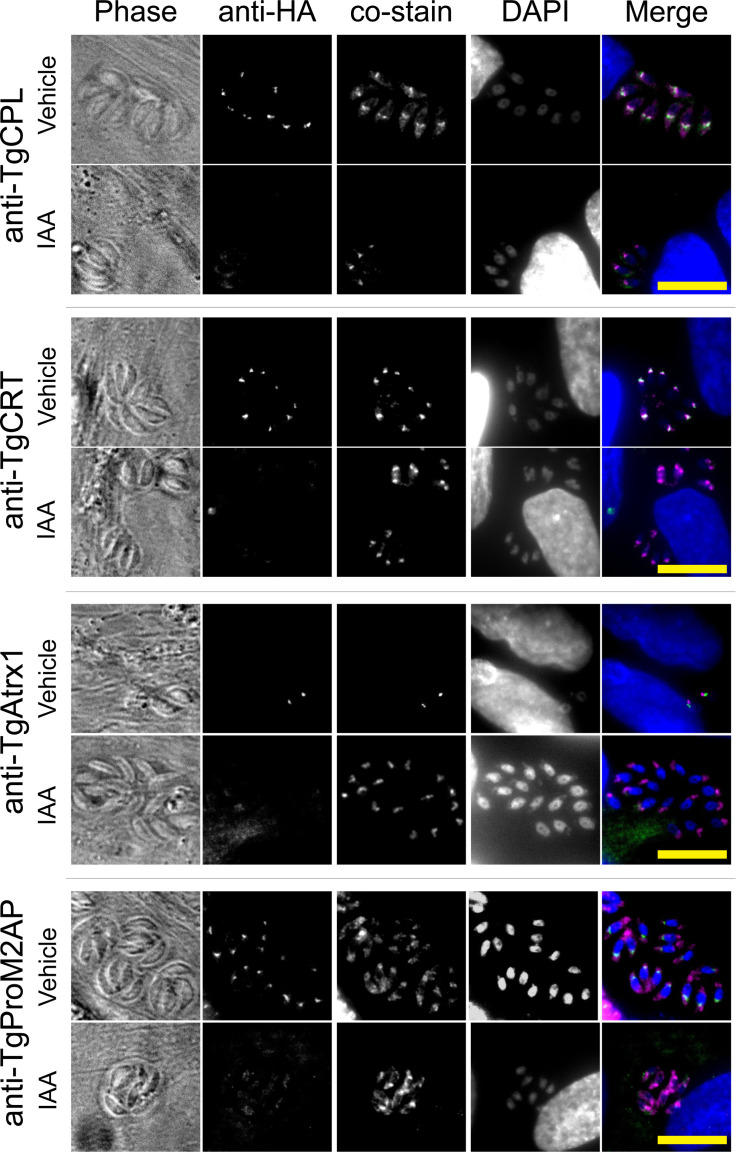
TgENT1 resides in the plant-like vacuolar compartment (PLVAC) and is eliminated by auxin-induced degradation. Representative immunofluorescence images of TgENT1-mAID-HA tachyzoites pre-treated for 48 h with vehicle or 500 µM +IAA, then grown in fresh Hs27 monolayers for a further 48 h under the same conditions. Parasites were fixed and stained with anti-HA (second column) plus one of the indicated compartment markers (third column): *T. gondii* cathepsin protease L (TgCPL) or *T. gondii* chloroquine-resistance transporter (TgCRT) for the PLVAC, TgAtrx1 for the apicoplast, and TgProM2AP for the endosome-like/microneme compartment. Columns show (left to right) phase-contrast, anti-HA, co-stain, DAPI, and the merged overlay (anti-HA = green, co-stain = magenta, and DAPI = blue). Under vehicle conditions, TgENT1-HA displays a punctate pattern that co-localizes with PLVAC markers TgCPL and TgCRT, but only weakly with TgProM2AP and not with TgAtrx1, consistent with predominant residence in PLVAC. Upon IAA treatment, the HA signal is virtually abolished, confirming efficient auxin-inducible degradation. Scale bar, 10 µm.

Using the TgENT1-mAID-HA construct, we were able to visualize the association of TgENT1 within *T. gondii* parasites. When cultured with 500 µM IAA, we observed the disappearance of the TgENT1 signal, confirming the system functionality (Fig. 5). Surprisingly, TgENT1 localization differed markedly from typical plasma membrane-associated ENTs. Instead, HA-TgENT1 displayed a distinctive punctate pattern within the parasite cytoplasm, suggesting that it is localized within intracellular organelles (Fig. 5).

To pinpoint the intracellular compartment that hosts TgENT1, we performed IFAs on parasites expressing HA-tagged TgENT1 alongside well-validated organelle markers. Co-staining was carried out with antibodies against *T. gondii* cathepsin protease L (TgCPL) and the chloroquine-resistance transporter (TgCRT), both definitive markers of the plant-like vacuole compartment (PLVAC) (27), as well as the propeptide of microneme protein 2 (TgProM2AP), which labels the endosome-like compartment (28), and an anti-apicoplast (TgAtrx1) antibody (29). Across all markers, in control conditions, the TgENT1 HA‐tagged protein shows partial colocalization with PLVAC markers: TgCPL [Pearson’s *r* ≈ 0.84; *M*(CPL) ≈ 0.90] and TgCRT [*r* ≈ 0.79; *M*(CRT) ≈ 0.54], weak overlap by the microneme protein TgProM2AP [*r* ≈ 0.61; *M*(M2AP) ≈ 0.09], and virtually no overlap with TgAtrx1. Here, *M*(*X*) represents Manders’ colocalization coefficients, which quantify the fraction of antibody *X*’s fluorescence that shares the same pixels as the HA signal (1.0 = complete overlap; 0 = none). These data indicate that, under control conditions, our protein of interest is predominantly localized to the parasite’s vacuolar system. Upon IAA‐induced knockdown (+IAA), colocalization with all antibodies declined, most dramatically for CRT [*M*(CRT) ≈ 0.02] and CPL [*M*(CPL) ≈0.27], further suggesting TgENT1 partial colocalization with PLVAC markers. Altogether, this points to the PLVAC as the principal compartment for TgENT1 protein.

### PLVAC measurement

**Fig 6:**
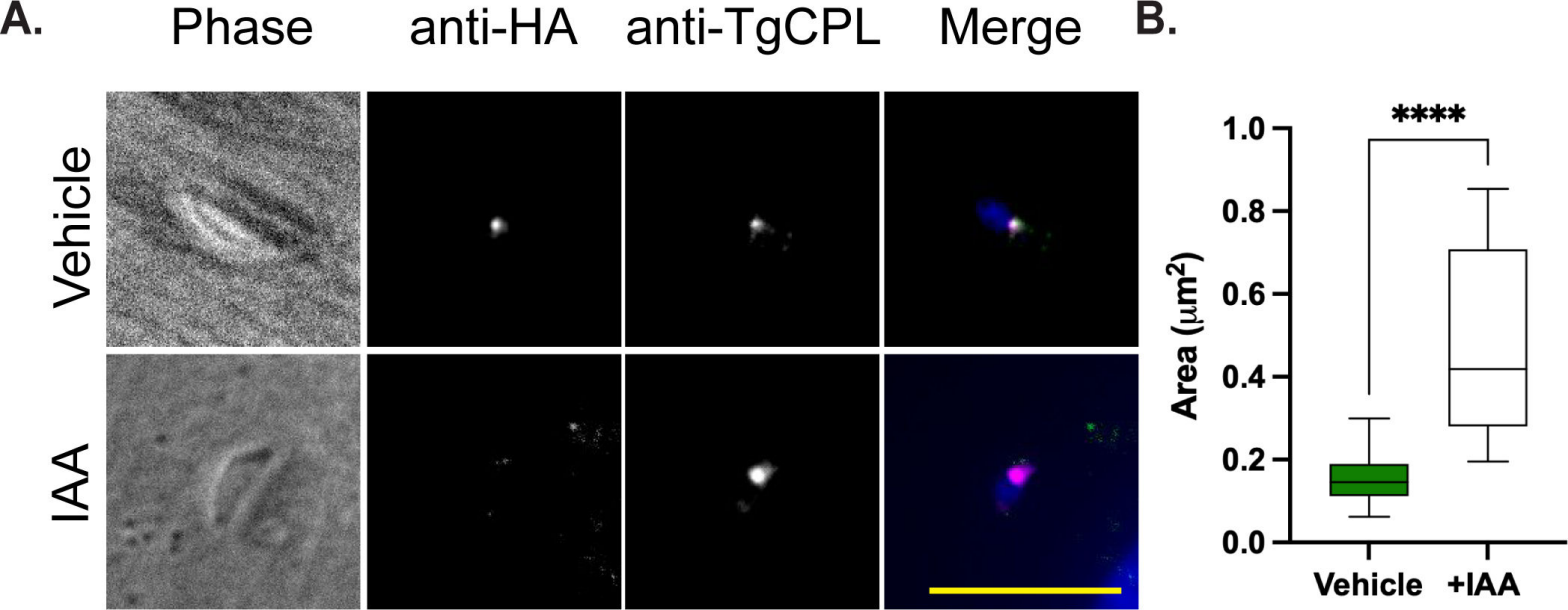
Loss of TgENT1 causes swelling of the PLVAC. (**A**) TgENT1-mAID-HA parasites were grown for 48 h post-infection, mechanically released, and allowed to reinvade new Hs27 monolayers for 30 minutes in the continued presence of vehicle or 500 µM IAA. Cells were fixed and co-stained with anti-HA (TgENT1, green) and anti-TgCPL (PLVAC marker, magenta); nuclei were counter-stained with DAPI (blue). TgENT1 is readily detected in the central PLVAC punctum under vehicle conditions but is efficiently depleted after auxin treatment, coincident with an enlarged TgCPL-positive compartment. Scale bar, 10 µm. (**B**) Quantification of PLVAC size. The area of 50 individual CPL-positive structures per condition (three independent experiments) was measured in FIJI. Box-and-whisker plots show median, interquartile range, and 5th–95th percentiles. Auxin-induced TgENT1 depletion increased PLVAC area nearly fivefold, Student *t*-test, ****P < 0.0001, suggesting that TgENT1 activity is required to maintain normal vacuolar homeostasis.

We evaluated PLVAC morphology following TgENT1 depletion by performing knockdown experiments in replicating parasites 36 h post-infection. Parasites were mechanically liberated and subsequently inoculated onto fresh Hs27 monolayers for 30 minutes before fixation and staining. Importantly, TgENT1 knockdown parasites retained their ability to invade host cells efficiently. Immunofluorescence assays were employed to measure PLVAC dimensions using an anti-TgCPL antibody (Fig. 6). The area from 50 TgCPL puncta per experimental condition was measured in triplicate, and comparative statistical analyses were performed using *t*-test. Consistent with our hypothesis, TgENT1 depletion resulted in measurable swelling of the PLVAC, further implicating TgENT1 as important for maintaining PLVAC homeostasis.

### Transcriptomic analysis of TgENT1 knockdown

**Fig 7:**
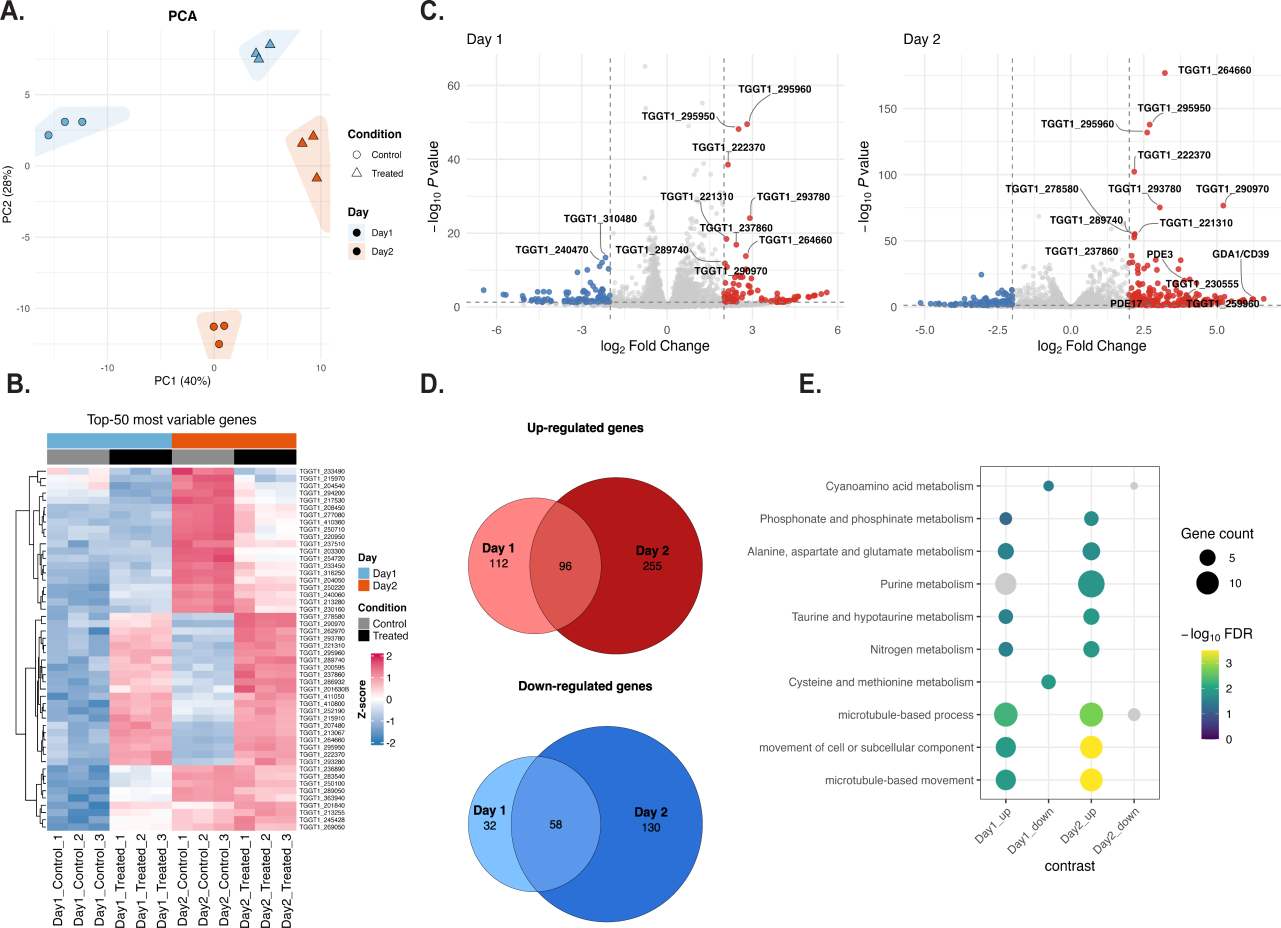
Transcriptomic response to acute TgENT1 depletion reveals a progressive, purine-centric stress program. TgENT1-mAID-HA tachyzoites (three biological replicates) were pre-treated for 48 h with vehicle or 500 µM IAA, then allowed to invade fresh Hs27 monolayers under the same conditions and harvested 24 h (day 1) or 48 h (day 2) after invasion. Poly(A) RNA was sequenced (Illumina NovaSeq; 150 bp PE), reads were mapped to the GT1 genome, and differential expression was analyzed with DESeq2 (|log₂FC| ≥ 1; false discovery rate [FDR] < 0.01). (**A**) Principal-component analysis (PCA). PC1 (40%) cleanly separates treated from control samples, whereas PC2 (28%) differentiates time points, indicating a robust, time-dependent transcriptional shift upon TgENT1 knockdown. (**B**) Heat map of the 50 most variable genes. *Z*-scored expression values highlight a concerted upregulation of purine- and nitrogen-metabolism genes (reds) and downregulation of cytoskeletal/motility genes (blues) that intensifies from day 1 to day 2. (**C**) Volcano plots of differentially expressed genes. Pink, upregulated; blue, downregulated; gray, non-significant. The magnitude and number of responsive genes expand markedly by day 2. The complete list of genes and fold change is presented in Table S4. (**D**) Overlap of DE genes between time points. Venn diagrams show 96 genes upregulated at both day 1 and day 2, and 58 genes consistently downregulated, underscoring a core TgENT1-dependent regulon that is sustained and amplified over time. (**E**) Functional enrichment analysis. Dot plot of the top gene ontology/KEGG terms (size = gene count; color = –log₁₀FDR). Upregulated sets are dominated by purine metabolism, nitrogen-compound metabolism, and cyano-/taurine-derivative pathways, whereas downregulated sets are enriched for microtubule-based movement and related processes, consistent with a shift from proliferation to metabolic stress response.

Transcriptomic profiling following conditional knockdown of TgENT1 revealed significant alterations in genes associated with purine metabolism, reinforcing TgENT1’s proposed function as a nucleoside transporter (Fig. 7). Principal-component analysis (Fig. 7A) cleanly separates IAA-treated and control tachyzoites along PC1 and shows a further time-dependent shift on PC2, underscoring a rapid and progressive transcriptional response to TgENT1 loss. An accompanying heat map (Fig. 7B) highlights the 50 most variable transcripts. Volcano plots of differentially expressed genes (Fig. 7C) display a broader and more pronounced response by 48 h, with the number and magnitude of regulated transcripts expanding and purine-stress markers (e.g., nucleoside phosphatases and phosphodiesterases [PDEs]) among the most strongly upregulated. For example, TGGT1_225290 (GDA1/CD39 nucleoside phosphatase) demonstrated progressive upregulation from day 1 (log₂ fold change = 2.98) to day 2 (log₂ fold change = 6.21). Similarly, TGGT1_259960 (putative nucleoside-diphosphatase) exhibited strong induction from day 1 (log₂ fold change = 2.81) to day 2 (log₂ fold change = 5.92), suggesting a sustained compensatory effort to restore purine homeostasis when nucleoside uptake is impaired. Importantly, other TgENT family members showed no significant expression changes.

In total, 208 transcripts were already altered at 24 h and this number more than doubled by 48 h, with 96 up- and 58 downregulated genes shared between both time points (Fig. 7D), defining a core TgENT1-dependent regulon. Among the induced genes were several 3’5’-cyclic nucleotide PDEs, notably PDE3 (TGGT1_233065; log₂ fold change = 1.30 at day 1, significantly increased to 3.88 at day 2), and PDE17 (TGGT1_257945; log₂ fold change = 2.92), suggesting heightened cyclic nucleotide turnover and signaling adaptation (30).

KEGG pathway analyses (Fig. 6E) revealed significant differential expression in pathways related to purine metabolism, nitrogen compound metabolism, and oxidoreductase activities. The enrichment of the purine metabolism pathway further intensified at day 2, according to KEGG pathway analysis (Fig. 7E; fold enrichment = 2.39, *P*-value = 0.0008), featuring upregulated genes like TGGT1_230555 (31) (adenylate/guanylate cyclase; log₂ fold change = 3.84) and further emphasizing signaling changes upon TgENT1 knockdown. Additionally, TGGT1_290970 (32) (8-amino-7-oxononanoate synthase), involved in biotin biosynthesis, was substantially induced from day 1 (log₂ fold change = 2.57) to day 2 (log₂ fold change = 5.22), suggesting enhanced biotin-dependent metabolic processes.

Collectively, these transcriptomic findings suggest TgENT1’s central role in purine transport and homeostasis within *T. gondii*. The extensive and progressive compensatory responses triggered by TgENT1 knockdown, including purine salvage, metabolic reprogramming, and signaling adaptations, provide new insights into the parasite’s ability to adapt to disrupted nucleoside transport.

## DISCUSSION

In this study, we expand the model of purine acquisition in *T. gondii* from a simple linear import pathway to a dynamic, regulated network. Notably, of the four homologs, only TgAT1 has been characterized *in vitro*; future work should define the precise substrate range and specificity of the remaining homologs. HyperLOPIT spatial-proteomics has previously assigned both TgAT1 and TgENT3 to the parasite plasma membrane (21) (Table 1). Although our experiments did not verify this localization directly, the functional data reveal a broader principle of metabolic resilience: *T. gondii* can rapidly re-wire its purine-uptake network to compensate for the loss of TgAT1 and TgENT3. Such plasticity is indispensable for an organism that is purine-auxotrophic and therefore wholly dependent on the host for nucleoside acquisition (33). Importantly, we also demonstrate that this adaptive capacity is limited during differentiation (Fig. 3). Understanding the mechanics, function, and ultimate failure point of this network provides unprecedented insight into the metabolic strategies governing the parasite’s lifecycle.

Our data show that deletion of the TgAT1 and TgENT3 triggers specific transcriptional upregulation of TgENT1 (Fig. 4A), a transporter that our localization studies partially co-localize in the PLVAC (Fig. 5). These findings support a model where the parasite mobilizes an alternative salvage pathway. This pathway might rely on acquiring purines from the degradation of host-derived nucleic acids within the acidic, digestive environment of the PLVAC (27) or from the recycling of its own nucleic acids, a function consistent with the PLVAC’s role in trafficking and catabolism (27). The adaptive function of the PLVAC and TgENT1 creates a crucial lifeline that sustains a baseline of purine homeostasis.

The failure of the ΔTgAT1ΔTgENT3 strain to develop as bradyzoites *in vitro* (Fig. 3A and C; Fig. S3) likely stems from two interconnected deficiencies: a substrate and a signaling deficit. The substrate deficit may deplete critical precursors for cyst formation, including the sugar-nucleotide donors uridine diphosphate N-acetyl-D-glucosamine (UDP-GlcNAc) and uridine diphosphate N-acetyl-D-galactosamine (UDP-GalNAc). These activated sugars supply N-acetylglucosamine and N-acetylgalactosamine residues that decorate cyst-wall glycoconjugates and are important for proper wall assembly (34). More critically, however, this purine starvation likely precipitates a failure in cellular signaling. Purine salvage is the sole source of the adenosine required for ATP synthesis. ATP, in turn, is the exclusive substrate for adenylyl cyclases that generate cyclic AMP (cAMP), a master second messenger in *T. gondii*. The cAMP signaling pathway is known to be an important regulator of stage conversion, with fluctuations in cAMP levels acting as a key signal for maintaining the tachyzoite state or initiating differentiation into bradyzoites (35). Therefore, we propose that the ΔTgAT1ΔTgENT3 strain, while receiving the external cues to differentiate (e.g., alkaline stress), is metabolically incapable of mounting the appropriate cAMP signaling response required to execute this complex developmental program. Interestingly, although ΔTgAT1ΔTgENT3 mutants form smaller cysts than parental strains in mice, the total cyst burden remains unchanged, suggesting that the 30-day *in vivo* period allows the parasites to overcome the differentiation delay observed *in vitro* (Fig. S4).

Our study illuminates a transcriptional and phenotypic signature of nucleoside starvation in the absence of TgENT1, and several open questions make the next experimental steps especially compelling. The robust upregulation of TgENT1 is currently inferred to reflect enhanced host-nucleoside scavenging; confirming this hypothesis with direct radiolabeled-substrate uptake assays will test our model and establish quantitative benchmarks for future transporter engineering. In addition, the nucleoside-depletion phenotype reported for Δgra14 parasites, which are defective in parasite endocytosis of material from the cytosol of infected cells, suggests that the PLVAC has a central role in nucleoside salvage and invites a systematic dissection of each vacuolar effector in that pathway (36). Finally, untargeted metabolomics coupled to parallel measurements of high-energy substrates such as ATP and GTP and their signaling derivatives would provide a systems-level view of how disrupted uptake reverberates through the parasite’s metabolic and regulatory networks.

## MATERIALS AND METHODS

### Parasite maintenance

*T. gondii* tachyzoites, ME49 and RH strains, were propagated in human foreskin fibroblasts (Hs27; ATCC CRL-1634) at 37°C in DMEM supplemented with 10% FBS, 100 U/mL penicillin, and 100 µg/mL streptomycin. Cells and parasite lines were tested for mycoplasma monthly using a PCR test (Boca Scientific).

### Plasmid construction

Final sequences are listed in Table S2. PCRs employed Q5 High-Fidelity (long fragments) or Taq DNA polymerase (routine amplifications). Amplicons and vector backbones were assembled with Gibson Assembly (NEB) unless noted:

- ΔTgENT3 donor—synthetic plasmid (GenScript);
- ΔTgENT3::TgENT3-Ty1 donor—23-DD plasmid digested with BglI, synthetic insert (B1) ordered and inserted (Twist Bioscience);
- ΔTgAT1 donor—ΔTgENT3 donor digested with AvrII/NotI (insert) and SpeI/AflII (backbone), 5′ and 3′ TgAT1 homology arms amplified with P1/P2 and P3/P4;
- TgENT3-DHFR-KO donor—DHFR cassette (Addgene #80329) amplified with P5/P6 and inserted into ΔTgENT3 backbone (NotI/EcoRV);
- mAID-HA-TgENT1 donor—synthetic plasmid (GenScript);
- ΔTgAT1::TgAT1-myc donor—24-DD plasmid digested with BssHI/ScaI, synthetic inserts (B2 and B3) ordered and inserted (Twist Bioscience);
- ΔTgENT2—ΔTgENT3 donor digested with AflII/SnaBI; synthetic insert (B4) ordered and inserted (Twist Bioscience);
- guide RNA plasmids—pSS013 vector (Addgene) was BsaI linearized, and annealed IDT oligos were inserted by Gibson Assembly, transformed into *E. coli* DH5α, sequence-verified, and prepared with GeneJET Maxi kits.

### Genomic editing and transfection

Knock-outs, knock-ins, and mAID tagging were generated with dual-guide CRISPR–Cas9 as described (voltage 1,500 V, 0.1 ms, two pulses; BTX ECM 830, 4 mm cuvette). Parasites harvested from confluent T25 flasks were washed once in cytomix (120 mM KCl, 0.15 mM CaCl_2_, 10 mM K_2_HPO_4_/KH_2_PO_4_ pH 7.6, 25 mM HEPES, 2 mM EGTA, and 5 mM MgCl_2_). Electroporation mixtures contained 50 µg linearized donor DNA and 25 µg of each sgRNA plasmid in 400 µL cytomix. Parasites were immediately added to fresh Hs27 monolayers. Drug selection began 24 h post-transfection (40 µM chloramphenicol or 3 µM pyrimethamine). Drug-resistant populations were single-cell-sorted (or cloned by limiting dilution), expanded, and validated by diagnostic PCR, western blot, and immunofluorescence (primer sets P7–P28; see Table S2).

### AlphaFold modeling

Predicted protein structures were retrieved from the AlphaFold Protein Structure Database (AlphaFold DB) using their UniProt accession code. These predicted structures were aligned with human ENT1 (PDBID: 6OB7) using PyMOL version 3.1.4.1 (Schrodinger). Root mean square deviation of each transporter relative to human ENT1 was calculated using PyMOL. Atomic measurements of pore width were also performed in PyMOL. As shown using HsENT1 in Fig. 1C, pore width was measured across the widest and narrowest points (yellow and red dashed lines). TgENT1, TgENT2, TgENT3, and TgAT1 pores were measured in the same manner.

### Immunofluorescence assay

Cells were fixed in 3.7% formaldehyde for 20 minutes, washed twice with 1× PBS, blocked, and permeabilized with 3% bovine serum albumin (BSA) and 0.2% Triton X-100 for 2 h at room temperature (or, alternatively, overnight at 4°C). Primary antibodies were diluted 1:500 (unless otherwise stated in Table S3) in blocking/permeabilization solution and incubated for 1 h at room temperature (or overnight at 4°C). Cells were washed twice with 1× PBS, applied with secondary antibodies (1:500 dilution; DAPI at 1:200) in blocking/permeabilization solution for 1 h at room temperature, washed three times for 5 minutes, and then mounted on glass coverslips and sealed.

### Manders’ colocalization coefficients

Manders’ coefficients measure the fractional overlap of signal intensities of two fluorophores after removing background. Manders’ M1 and M2 report what fraction of fluor A lies where fluor B is present (and vice versa), independent of proportionality. Colocalization was quantified in raw 16-bit TIFF images using Fiji (ImageJ) Coloc 2. After identical acquisition (no saturation, sequential capture to avoid bleed-through), images were opened without prior gamma adjustments, and a uniform rolling-ball background subtraction was applied to both channels; no nonlinear filtering or manual thresholding was performed. Reported values are the mean of at least 50 vacuoles.

### Bradyzoite *in vitro* differentiation

The *in vitro* differentiation was performed as previously (35). Confluent Hs27 fibroblast monolayers on glass coverslips were infected with freshly egressed tachyzoites (multiplicity of infection [MOI] ≈ 1; 2 h invasion + gentle wash; total pre-induction incubation 4 h). Cultures were then shifted to pre-warmed alkaline induction medium (RPMI 1640 supplemented with 1% heat-inactivated FBS, 50 mM HEPES, pH 8.20 ± 0.05 at 37°C) and incubated in ambient air (≈0.04% CO_2_) in a humidified chamber (no external CO_2_) to maintain pH ≥ 8.0. Induction medium was replaced every 24 h with freshly adjusted medium; pH was spot-checked at each change. After 2, 3, or 7 days post-infection (dpi), coverslips were fixed (4% paraformaldehyde/PBS, 20 min), permeabilized (0.2% Triton X-100), and dual stained with rabbit anti-*Toxoplasma* antibody (Thermo Fisher; 1:500) to delineate vacuoles and DBA (2–5 µg/mL) to label the cyst wall. A vacuole was scored “differentiated” if ≥75% of its perimeter showed a continuous DBA rim. For each condition, ≥100 vacuoles per coverslip were scored blindly (≥3 biological replicates).

### Tachyzoite intracellular growth and plaque assays

Confluent Hs27 (human foreskin fibroblast) monolayers on glass coverslips were infected with each *T. gondii* strain at an MOI yielding mostly isolated vacuoles (typically MOI 0.5–1.0) in standard pH 7.4 medium (DMEM + 10% FBS, 5% CO_2_). After allowing invasion for 2 h at 37°C, extracellular parasites were removed by two PBS washes, and fresh medium was added. At the indicated post-infection time point in each experiment, cells were fixed in 4% paraformaldehyde (15 min), permeabilized (0.1% Triton X-100, 10 min), and blocked (3% BSA/PBS, 30 min). Parasites were labeled with anti-*Toxoplasma* (followed by Alexa Fluor–conjugated secondary antibody). For each biological replicate, 100 intact, non-overlapping parasitophorous vacuoles were randomly selected using a Cytation 7 imager (Agilent) under consistent exposure settings, and the number of tachyzoites per vacuole was recorded. Three biological replicates were performed per experiment. Vacuoles at the edge of the field, those that were obviously disrupted, or those with uncertain boundaries were excluded from counting.

For plaque assays, confluent HS27 monolayers in six-well plates were infected with 1,000 freshly egressed tachyzoites in 2 mL of complete medium. At the endpoint, monolayers were fixed in 100% ethanol (10 min) and stained with 0.1% crystal violet (in 20% methanol) for 15–20 min, rinsed, and air-dried. Plaques (zones of host cell clearance) were imaged and counted manually, and plaque areas were measured after background subtraction and uniform threshold application. At least three biological replicates (independent parasite preparations on different days) per strain were analyzed. Statistical analysis used one-way ANOVA with appropriate multiple comparison correction. Quality controls: parasite viability and inoculum accuracy were confirmed by counting extracellular tachyzoites with a hemocytometer.

### RNA extraction and mRNA sequencing

Total RNA was extracted from *T. gondii*-infected cells using the GeneJET RNA Purification Kit (Thermo Fisher Scientific) according to the manufacturer’s protocol. RNA quality and concentration were confirmed, and poly(A)-selected mRNA libraries were subsequently prepared by Novogene. Libraries were sequenced on an Illumina NovaSeq X Plus platform, generating 150 bp paired-end reads. All experiments were performed in biological triplicate.

### Immunofluorescence assay for PLVAC morphology

PLVAC morphology was assessed 36 h post-infection in TgENT1 AID-HA tachyzoites ± IAA. Parasites were mechanically released (27 g needle) from host cells and immediately inoculated onto fresh confluent Hs27 monolayers for a 30 min invasion pulse (37°C). Cultures were fixed in 4% paraformaldehyde (20 min, RT) and stained with primary anti-TgCPL antibody (PLVAC marker) followed by appropriate fluorophore-conjugated secondary antibodies and DAPI. Imaging was performed under identical acquisition settings across conditions. For each condition, the cross-sectional area of the first 50 TgCPL-positive PLVAC puncta associated with intracellular parasites was quantified (Fiji) in each of three biological replicates (*n* = 50 vacuoles/condition). Areas were compared by *t*-test.

### Animal experiments

Mice were housed under a 12 h light/12 h dark cycle with daily health monitoring that began 1 week before infection and continued until study end; animals were euthanized if predefined humane endpoints were met.

Two independent experiments were conducted. In each, four adult male CBA/J mice (The Jackson Laboratory) per parasite strain received an intraperitoneal inoculation of 250 freshly egressed tachyzoites suspended in 200 µL sterile PBS. At 30 dpi, mice were euthanized following IACUC protocol, and whole cortices were promptly harvested for downstream analyses.

Brain immunofluorescence was conducted as previously reported (37). Briefly, the tissue was homogenized using serial needles passing through 18-, 20-, and 22-gage needles, pelleted at 1,000 × *g* for 5 minutes, and fixed with 3.7% formaldehyde for 20 minutes. Pellets were washed twice with 1× PBS and blocked overnight in 1% BSA and 0.2% Triton X-100 in 1× PBS. Primary antibodies diluted 1:500 were incubated overnight, washed twice with 1× PBS, and secondary antibodies (1:500 dilution) and DAPI were added for 1 h, washed twice with 1× PBS, and imaged using a microscope.

Cysts stained with DBA were wet mounted onto coverslips and scanned for DBA using a Cytation 7 imager (Agilent). Fifty randomly selected cysts per sample were measured and recorded using Agilent BioTek Gen5 software.

### Quantitative PCR

RNA was extracted from freshly lysed tachyzoites using the GeneJet RNA Purification Kit. cDNA was subsequently synthesized employing the Maxima H Minus First Strand cDNA Synthesis Kit. qPCRs were then set up with primers Q1 – Q8 using the PowerSYBR Green PCR Master Mix.

### Statistical analysis

GraphPad Prism 9 was used for all statistics. Data are mean ± SEM of ≥3 independent experiments. Group differences were analyzed by one-way ANOVA or unpaired two-tailed Student’s *t*-test (as indicated). *P* < 0.05 (*), 0.01 (**), and 0.0001 (****) were considered significant.

## Data Availability

Data are publicly available in the NCBI Sequence Read Archive under BioProject accession PRJNA1247533.

## SUPPLEMENTAL MATERIAL

Fig. S1 mbio.02207-25-s0001.tif Multiple sequence alignment.

Fig. S2 mbio.02207-25-s0002.tif Intracellular replication and multi-cycle lytic growth of parental and ENT mutant strains.

Fig. S3 mbio.02207-25-s0003.tif Reduced cyst size from *in vivo* differentiated ΔTgENT3 and ΔTgAT1ΔTgENT3 relative to parental and single ENT mutants.

Fig. S4 mbio.02207-25-s0004.tif *In vivo* cyst quantification.

Fig. S5 mbio.02207-25-s0005.tif PCR validation of TgENT knockouts, double knockout, and epitope/complementation lines.

Legends mbio.02207-25-s0006.docx Legends for supplemental material.

Table S1 mbio.02207-25-s0007.xlsx IC₅₀ of Ara A and 5 FU in *Toxoplasma gondii* strains.

Table S2 mbio.02207-25-s0008.xlsx Oligonucleotide list detailing ssDNA sequences used for cloning, genotyping, qPCR, guide RNAs, and plasmids.

Table S3 mbio.02207-25-s0009.xlsx Antibodies used for immunofluorescence detection.

Table S4 mbio.02207-25-s0010.xlsx Differentiation expression analyses of TgENT1 knockdown 24 and 48 h post-infection.

ASM does not own the copyrights to Supplemental Material that may be linked to, or accessed through, an article. The authors have granted ASM a non-exclusive, world-wide license to publish the Supplemental Material files. Please contact the corresponding author directly for reuse.
