# Supplementary figures and images for: Impact of equilibrative nucleoside transporters on Toxoplasma gondii infection and differentiation
Source: mBio. 2025 Sep 30;16(11):e02207-25. doi: 10.1128/mbio.02207-25 (PMC12607627; doi:10.1128/mbio.02207-25)

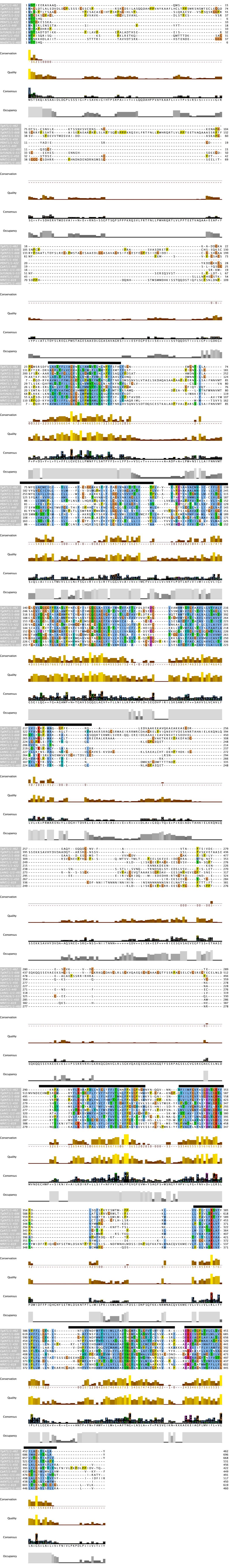

Supplement: Fig. S1 — Multiple sequence alignment. [file mbio.02207-25-s0001.tif]

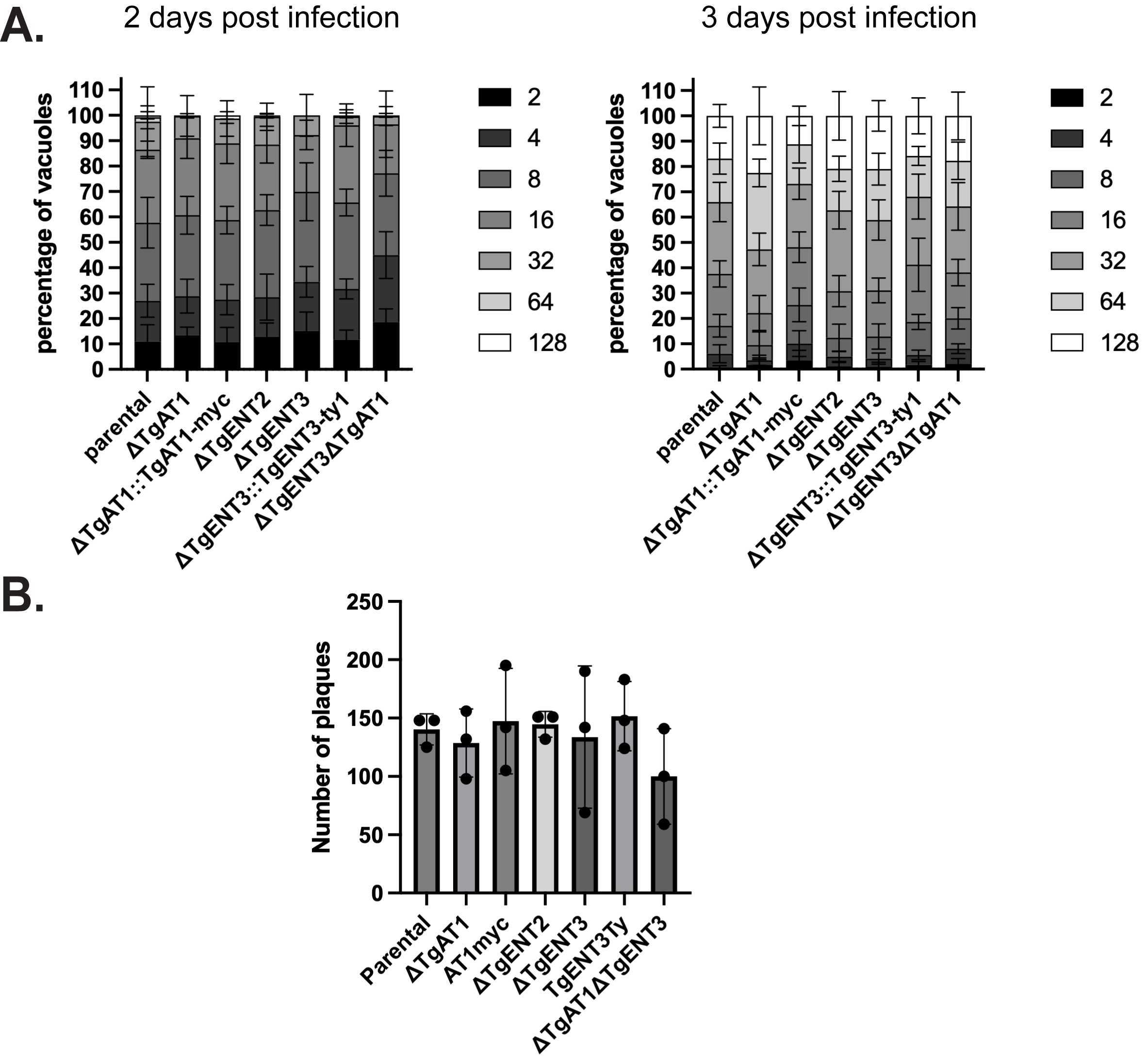

Supplement: Fig. S2 — Intracellular replication and multi-cycle lytic growth of parental and ENT mutant strains. [file mbio.02207-25-s0002.tif]

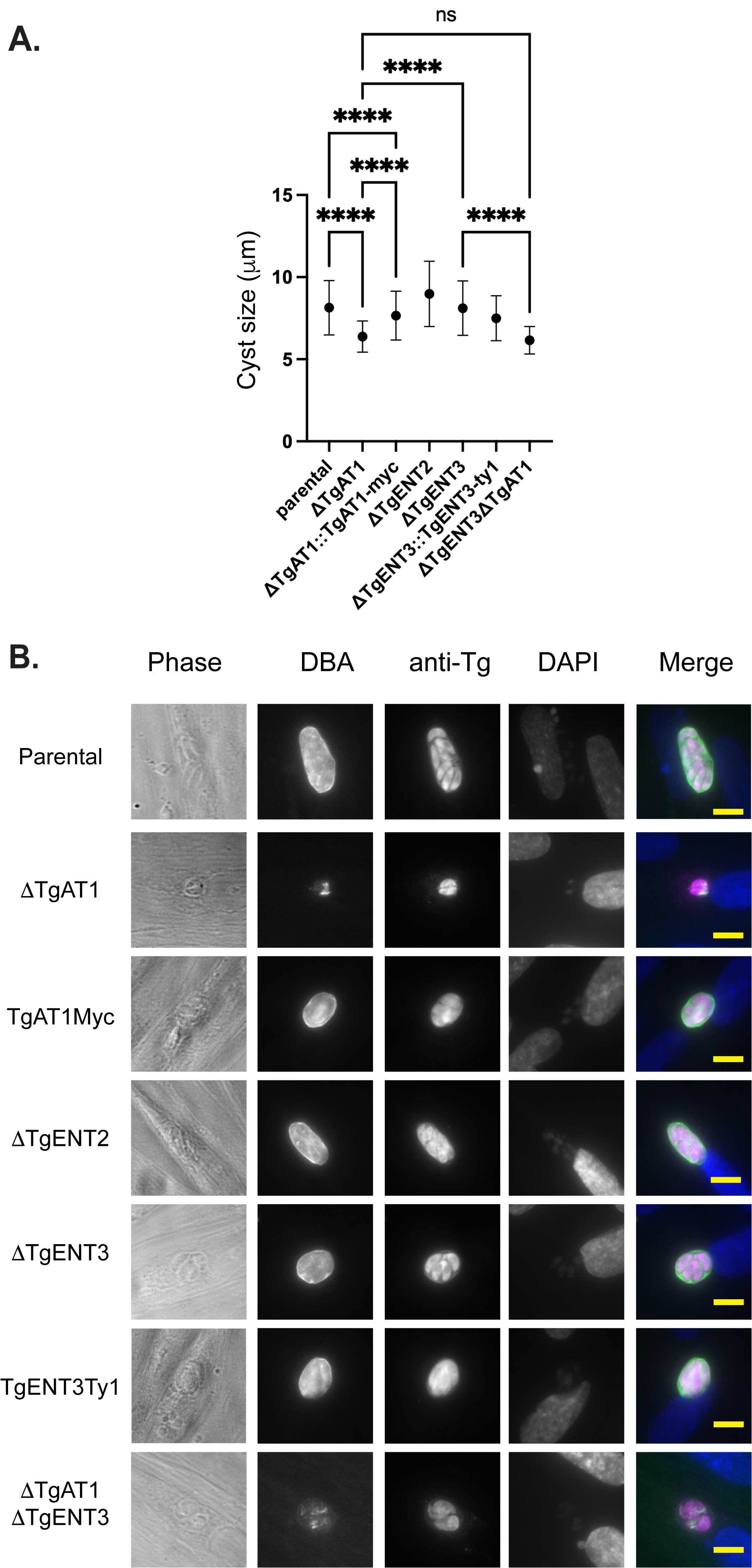

Supplement: Fig. S3 — Reduced cyst size from in vivo differentiated ΔTgENT3 and ΔTgAT1ΔTgENT3 relative to parental and single ENT mutants. [file mbio.02207-25-s0003.tif]

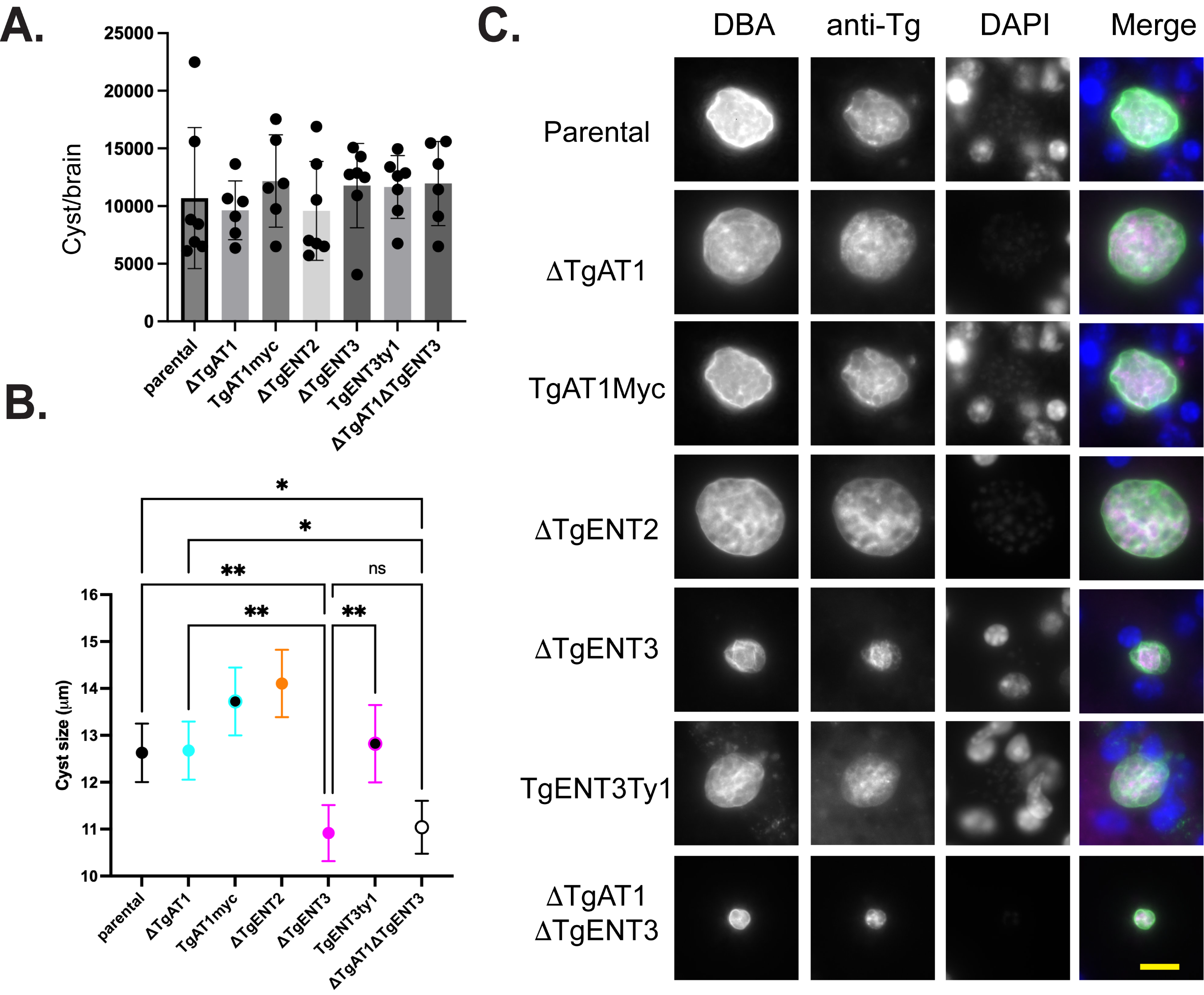

Supplement: Fig. S4 — In vivo cyst quantification. [file mbio.02207-25-s0004.tif]

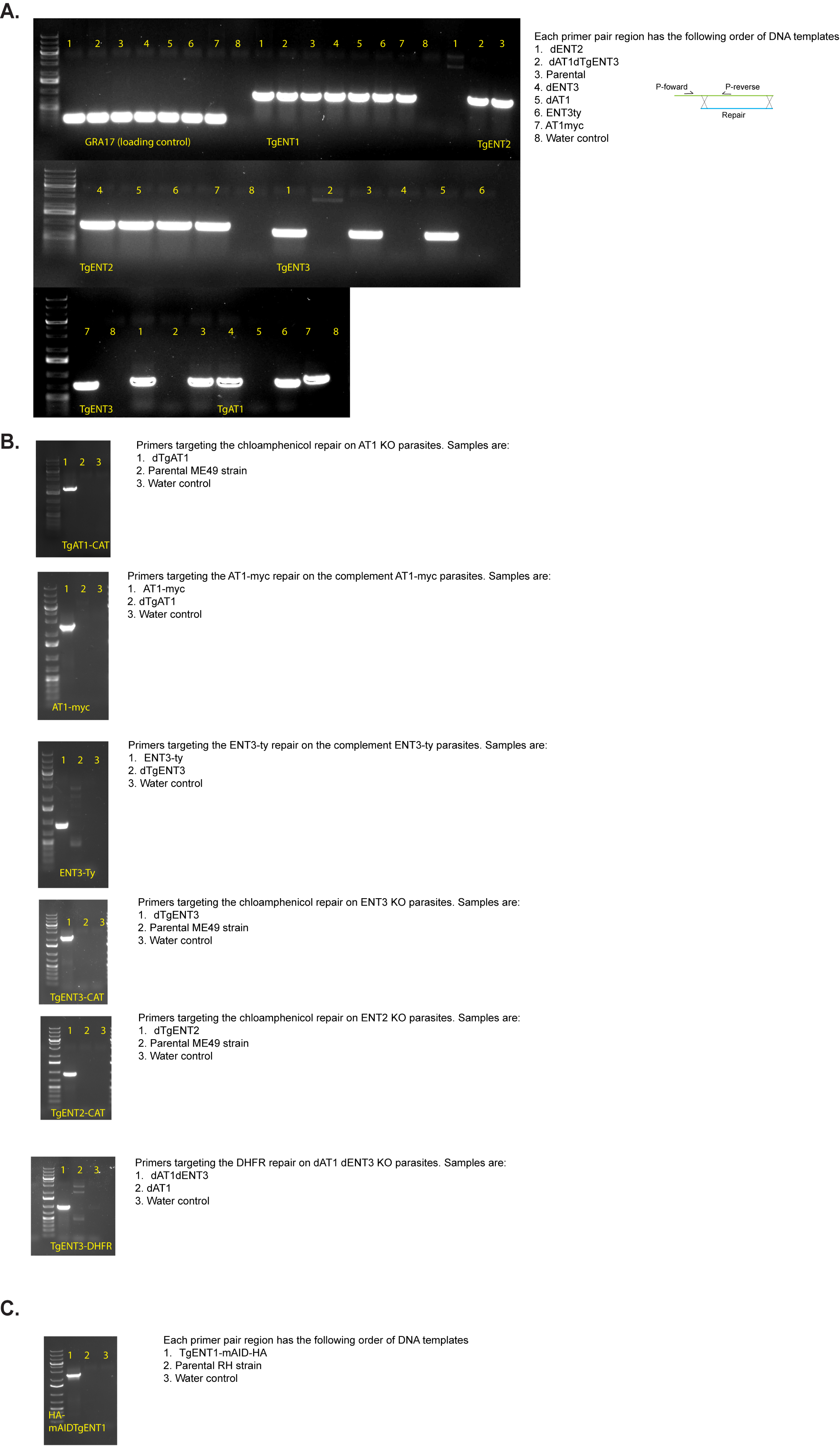

Supplement: Fig. S5 — PCR validation of TgENT knockouts, double knockout, and epitope/complementation lines. [file mbio.02207-25-s0005.tif]
