# Supplementary material for: Impact of equilibrative nucleoside transporters on Toxoplasma gondii infection and differentiation
Source: mBio. 2025 Sep 30;16(11):e02207-25. doi: 10.1128/mbio.02207-25 (PMC12607627; doi:10.1128/mbio.02207-25)
Supplement: Legends — for supplemental material. [file mbio.02207-25-s0006.docx]

**Supplemental material:**

**Supplemental Figure 1: Multiple sequence alignment of equilibrative nucleoside transporter (ENT/AT) homologs highlighting conservation across apicomplexan, kinetoplastid, and human sequences, with human ENT1 transmembrane topology reference.**Shown are *Toxoplasma gondii* TgAT1 (TGME49_244440), TgENT1 (TGME49_288540), TgENT2 (TGME49_500147), TgENT3 (TGME49_233130); *Plasmodium falciparum* PfENT1; *Cryptosporidium parvum* CpNT1; *Leishmania major* LmNT1; *Saccharomyces cerevisiae* FUN26; *Arabidopsis thaliana* ENT1; *Naegleria fowleri* NT1-like; and *Mus musculus* ENT1, and human ENT1. Sequence labels list protein shorthand (species in parentheses omitted for concision here) followed by aligned residue span (e.g., "/1–696"). Sequences were aligned with MAFFT L-INS-i; no manual internal residue edits were introduced (only removal of obvious trailing low-complexity overhangs where noted). Amino acids are colored by physicochemical class (ClustalX scheme) to facilitate rapid visual inspection of conserved motifs. Histogram bars beneath each block depict position-specific conservation (gold = high conservation / strongly similar physicochemical class; gray = variable) and gap frequency (black). Black horizontal rectangles above the alignment mark the experimentally validated transmembrane helices of human ENT1 (SLC29A1), provided as a structural topology reference against which parasite, plant, protozoan, and kinetoplastid homologs can be compared.

**Supplemental Figure 2: Intracellular replication (parasites per vacuole) and multi-cycle lytic growth (plaque numbers) of parental and ENT mutant strains.**
**(A)** Distribution of vacuole sizes (stacked categories: 2, 4, 8, 16, 32, 64, 128 parasites/vacuole) at 2 days (left) and 3 days (right) post-infection under standard tachyzoite conditions for parental ME49Δku80, ΔTgAT1, complemented ΔTgAT1::TgAT1-myc, ΔTgENT2, ΔTgENT3, complemented ΔTgENT3::TgENT3-ty, and the double mutant ΔTgAT1ΔTgENT3. For each strain and time point at least 100 vacuoles were scored per biological replicate (n=3 independent infections). Bars show mean percentage ± SEM of total vacuoles; categories sum to ~100%. No significant differences in overall replication distribution between strains were detected by two-way ANOVA (factors: strain, vacuole size category) (strain comparisons at each time point). (**B)** Plaque formation after 10 days of continuous lytic growth in Hs27 monolayers (crystal violet staining). Bars indicate mean plaque number ± SEM (n=3 independent plates per strain; each point = one plate). One-way ANOVA revealed no significant difference among strains (p>0.05). Data indicate that deletion (or complementation) of individual ENTs or combined loss of TgAT1 and TgENT3 does not measurably impair tachyzoite intracellular replication over 72 h nor cumulative multi-cycle growth under these assay conditions.

**Supplementary Figure S3. Reduced cyst size in ΔTgAT1 and ΔTgAT1ΔTgENT3 relative to parental and single ENT mutants.** **(A)** Quantification of cyst diameters (median ± Standard Error of the Mean) for in vitro differentiated bradyzoites (alkaline pH, Day 3) of the indicated strains: parental ME49Δku80, ΔTgAT1, complemented ΔTgAT1::TgAT1-myc, ΔTgENT2, ΔTgENT3, complemented ΔTgENT3::TgENT3-ty, and the double mutant ΔTgAT1ΔTgENT3. Each point represents the mean of one biological replicate (n=3 independent differentiations); ≥150 DBA+ cysts were measured per strain per replicate using an automated ImageJ macro**.** One-way ANOVA multiple comparisons test detected significantly smaller cysts in ΔTgAT1 and ΔTgAT1ΔTgENT3 compared with parental and respective complemented strains (**** p<0.0001); "ns" = not significant. **(B)** Representative high-magnification images of DBA-stained cysts (magenta in merge; direct grayscale shown in second column) from each strain at Day 3. Columns: transmitted light (Phase), DBA (cyst wall), anti-Tg (pan-parasite antibody marking internal bradyzoites), DAPI (host and parasite nuclei), and merged composite (DBA green, anti-Tg magenta, DAPI blue). Complemented lines restore cyst sizes and wall morphology towards parental phenotype. Scale bars: 10 µm. Images acquired under identical exposure and processing settings across strains. Data support a delay/reduction in cyst maturation rather than a complete differentiation block in transporter mutants.

**Supplementary Figure S4. In vivo brain cyst burden and morphology after 30 days of infection with parental and ENT mutant strains. (A)** Total brain cyst counts (cysts per mouse) at 30 days post intraperitoneal infection (250 tachyzoites) with the indicated strains: parental ME49Δku80, ΔTgAT1, complemented ΔTgAT1::TgAT1-myc, ΔTgENT2, ΔTgENT3, complemented ΔTgENT3::TgENT3-ty, and the double mutant ΔTgAT1ΔTgENT3. Each point = one mouse brain; bars show mean ± SEM. Two independent experiments were performed (4 male C57BL/6J mice per strain per experiment; symbols pooled after confirming no experiment–strain interaction by two-way ANOVA). No significant difference in total cyst burden among strains (one-way ANOVA, p>0.05). **(B)** Mean cyst diameter (µm) ± Standard Error of the Mean (SEM) for DBA-positive cysts from the same brains (≥50 cysts measured per strain across mice; automated ImageJ pipeline with uniform thresholding). ΔTgENT3 and ΔTgAT1ΔTgENT3 display significantly smaller cysts relative to parental and their respective complemented lines (one-way ANOVA with Tukey's multiple comparisons). *p<0.05, **p<0.01, ns = not significant.
**(C)** Representative fluorescence images of individual brain cysts stained with Dolichos biflorus agglutinin (DBA; cyst wall, green in merge), anti-Toxoplasma (pan-parasite antibody, magenta), and DAPI (nuclei, blue). Smaller, less fully expanded cysts are evident in ΔTgENT3 and most pronounced in the double mutant. Complementation (TgENT3-ty1) restores cyst morphology toward parental appearance. Images acquired under identical acquisition settings; Scale bar: 10 µm.

**Supplementary Figure S5. PCR validation of TgENT knockouts, double knockout, and epitope/complementation lines.**

**(A) Diagnostic PCR across endogenous loci.** Genomic DNA from eight parasite lines (lane order, left to right: 1 ΔTgENT2; 2 ΔTgAT1ΔTgENT3; 3 parental ME49; 4 ΔTgENT3; 5 ΔTgAT1; 6 ΔTgENT3::TgENT3-Ty1; 7 ΔTgAT1::AT1-myc; 8 H₂O control) was amplified with primer pairs specific for GRA17 (loading control) (P7/P8), TgENT1 (P9/P10), TgENT2 (P11/P12), TgENT3 (P13/P14) or TgAT1 (P15/P16), as indicated above each gel strip. Absence of the wild-type amplicon (or size-shift where a repair cassette was inserted) confirms successful disruption, whereas parent and complemented strains retain the expected band.

**(B) Verification of selectable-marker and complementation inserts.**
Individual PCRs using primers flanking the repair junctions demonstrate correct integration of the chloramphenicol-acetyl-transferase (CAT) cassette into ΔTgAT1 (top) (P17/P18) and ΔTgENT3 (fourth panel) (P19/P20), of the AT1-myc (P21/P22) and ENT3-Ty (P23/P24) complementation cassettes into their respective loci (second and third panels), and of a DHFR cassette into the ΔTgAT1ΔTgENT3 line (bottom) (P25/P26). Lane order for each gel: 1 edited line; 2 parental ME49; 3 water control. The PCR product was Sanger-sequenced as confirmation.

**(C) Integration of the mAID-HA tag at the TgENT1 locus.**
PCR with locus-specific primers yields the diagnostic HA-junction product exclusively in the TgENT1-mAID-HA strain (lane 1) but not in RH-TIR1 (lane 2) or water control (lane 3), confirming single-locus tagging (P27/P28).

Molecular-weight ladders are shown in the first lane of each gel; expected band sizes are indicated by yellow arrowheads. Together, these assays verify the correct genetic architecture of all strains used in this study.

**Supplemental Table 1:** IC₅₀ of Ara‑A and 5‑FU in *Toxoplasma gondii* strains**.** Half‑maximal inhibitory concentrations (IC₅₀, µM) were measured for the parental line and mutants ΔTgAT1, AT1myc, ΔTgENT2, ΔTgENT3, TgENT3‑Ty, and ΔTgAT1ΔTgENT3. For each strain, the IC₅₀ point estimate is shown together with the observed range and the 95% confidence interval (CI). Ara‑A, vidarabine; 5‑FU, 5‑fluorouracil.

**Supplemental Table 2:** Oligo list detailing ssDNA sequences used for cloning, genotyping, qPCR, and guide RNAs, and DNA sequences of used plasmids.

**Supplemental Table 3**: List of antibodies used for immunofluorescence detection.

**Supplemental Table 4:** Differentiation expression analyzes of TgENT1 knockdown 24- and 48-hours post-infection.
